# Protein molecular modeling shows residue T599 is critical to wild-type function of POLG and description of a novel variant associated with the SANDO phenotype

**DOI:** 10.1038/hgv.2018.16

**Published:** 2018-04-05

**Authors:** John E Richter, Hector G Robles, Elizabeth Mauricio, Ahmed Mohammad, Paldeep S Atwal, Thomas R Caulfield

**Affiliations:** 1 Department of Clinical Genomics, Mayo Clinic, Jacksonville, FL, USA; 2 Department of Radiology, Mayo Clinic, Jacksonville, FL, USA; 3 Department of Neurology, Mayo Clinic, Jacksonville, FL, USA; 4 Department of Neuroscience, Mayo Clinic, Jacksonville, FL, USA; 5 Mayo Graduate School, Neurobiology of Disease, Mayo Clinic, Jacksonville, FL, USA; 6 Department of Health Sciences Research, Mayo Clinic, Jacksonville, FL, USA

## Abstract

Sensory ataxic neuropathy with dysarthria and ophthalmoparesis (SANDO) is a rare phenotype resulting from pathogenic variants of mitochondrial DNA polymerase gamma (*POLG*). We modeled a novel *POLG* variant, T599P, that causes the SANDO phenotype and another variant at the same residue, p.T599E, to observe their effect on protein function and confirm the pathogenicity of T599P. Through neoteric molecular modeling techniques, we show that changes at the T599 residue position introduce extra rigidity into the surrounding helix–loop–helix, which places steric pressure on nearby nucleotides. We also provide a clinical description of the T599P variant, which was found in a 42-year-old female proband. The proband presented a 1-year history of progressive gait instability, dysarthria and foot numbness. Her neurologic examination revealed ataxic dysarthria, restricted eye movements, head and palatal tremors, reduced lower limb reflexes, distal multimodal sensory loss and a wide, unsteady ataxic gait. Electromyography studies indicated a sensory neuropathy. Whole-exome sequencing was pursued after tests for infectious, inflammatory and paraneoplastic causes were negative.

## Introduction

An array of mitochondrial disorders has been linked to pathogenic variants in *POLG*, which encodes mitochondrial DNA (mtDNA) polymerase gamma (POLG). The integrity of this protein is crucial to mtDNA stability and replication, as pathogenic variants often cause mtDNA deletions or depletions.^1^ *POLG* pathogenic variants have been associated with several disease phenotypes, including myoclonic epilepsy myopathy sensory ataxia, autosomal recessive and dominant forms of progressive external ophthalmoplegia, and the ataxia neuropathic spectrum.^2^ The ataxia neuropathic spectrum includes sensory ataxic neuropathy with dysarthria and ophthalmoparesis (SANDO), a rare phenotype that has only been recorded in a small number of clinical cases.^1^

mtDNA depletion in both skeletal muscle and peripheral nerve tissue stems from certain pathogenic variants of *POLG*, resulting in dysfunctional mitochondria and the clinical triad of sensory ataxic neuropathy, dysarthria and ophthalmoparesis.^3^ In this report, we model a novel *POLG* variant that causes a SANDO phenotype, T599P, and another variant at the same residue, p.T599E, to observe their effect on protein function and to confirm the pathogenicity of T599P. We describe a proband with the T599P variant found in the linker domain (amino acid residues 418–755) of *POLG*.^4^ We propose that this pathogenic variant correlates with the SANDO condition.

## Materials and methods

Using genomic DNA from the submitted specimen, the exonic regions and flanking splice junctions were captured using a proprietary system developed by GeneDx and then were sequenced by massively parallel (NextGen) sequencing on an Illumina system with 100 bp or greater paired-end reads. Reads were aligned to the human genome build GRCh37/UCSC hg19 and analyzed for sequence variants using a custom analysis tool (Xome Analyzer). Capillary sequencing or another appropriate method was used to confirm all potentially pathogenic variants identified in this individual. Sequence alterations were reported according to the Human Genome Variation Society nomenclature guidelines.

The entire mitochondrial genome from the submitted sample was amplified and sequenced using a solid-state sequencing by-synthesis process. The DNA sequence was assembled and analyzed in comparison to the revised Cambridge Reference Sequence and the reported variants and polymorphisms listed in the MITOMAP database (http://www.mitomap.org). The presence of a disease-associated sequence variant was confirmed by conventional dideoxy sequence analysis or other methods. A reference library of more than 6,000 samples from different ethnic groups and online databases of mtDNA variations was used to evaluate variants of unknown clinical significance.

The sequence of human DNA polymerase subunit gamma-1 (POLG), a protein encoded by *POLG*, was taken from the NCBI Reference Sequence NM_002693.2: NP_002684.1 and used for computer-assisted modeling. Monte Carlo simulations were performed on the mutant to allow local regional changes after the p.T599P and p.T599E variants were introduced.

The X-ray refinement for Monte Carlo was built using the YASARA SSP/PSSM method.^5–10^ The structure was relaxed to the YASARA/Amber force field using knowledge-based potentials within YASARA. The side chains and rotamers were adjusted with knowledge-based potentials, simulated annealing with an explicit solvent and small equilibration simulations using YASARA’s refinement protocol.^11^ The entire full-length structure was modeled, and gaps or unresolved portions were filled from the X-ray.

Refinement of the finalized models was completed using either Schrodinger’s LC-MOD Monte Carlo-based module or NAMD2 protocols. These refinements started with an initial refinement and the mutants T599P and T599E, generated by YASARA.^5–7,9^ The superposition and subsequent refinement of the overlapping regions yielded a complete model of POLG. The final structures were subjected to energy optimization with a PR conjugate gradient with an R-dependent dielectric.

The atom consistency was checked for all 1,239 amino acids to verify the correct chain name, dihedrals, angles, torsions, non-bonds, electrostatics, atom-typing and parameters. Each model was exported to the following programs: Maestro (MAE); and YASARA (PDB). Model manipulation was done with Maestro (Macromodel, version 9.8, Schrodinger, LLC, New York, NY, USA) or Visual Molecular Dynamics.^12^

Monte Carlo dynamics searching (LCMOD-MC) was completed on each model for conformational sampling using methods described in the literature.^13–16^ Briefly, each POLG variant system was minimized with relaxed restraints using either Steepest Descent or Conjugate Gradient PR then allowed to undergo the Monte Carlo search criteria, as described in the literature.^13–16^ The primary purpose of Monte Carlo was to examine the conformational variability from different mutations in the region near the mutation and to analyze the possible effects of POLG on DNA binding or processing.

## Results

### Molecular modeling

**Figure 1:**
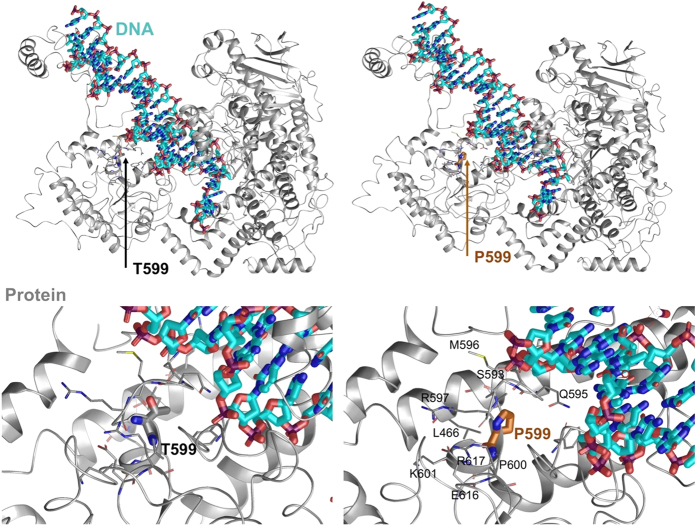
Schematic representation (three-dimensional molecular representation) of DNA polymerase gamma (POLG) side chain interactions within the local region and the T599P variant as well as its effect on DNA enzymatic processes. DNA is shown with cyan carbons and POLG with gray carbons.

For p.T599P, we found the energetic ΔΔ*G* shift to be +0.401813 kcal/mol Å^2^, which would increase the energy within the local region compared to the wild type.^13–15,17–20^ Local residues within the 6 Å cutoff under the influence of the mutation include Leu466, Ser592, Gln595, Met596, Arg597, Pro600, Lys601, Glu616, Arg617 and Phe766. The effects of the energetic changes included increased rigidity and directional reorientation of the loop segment from the loop–helix–loop, which promoted interactions between Gln595 and the phenylalanines adjacent to the DNA-binding regions. The entire region changes in root mean square deviation of the side chain atoms was 4.3813 Å. The maximum difference between atom pairs for the phenylalanine (Phe766) ring atoms was 13.7707 Å, although Gln595 was significantly altered. Gln595 and Phe766 may significantly alter DNA-binding efficiency, whereas the double prolines (Pro599 and Pro600) changed the orientation of the loop between the helices shown (Figure 1). Thus, the reoriented side chains may weaken or alter the process of DNA binding, whereas “rigidifying” the loop may render it less able to accommodate induced fit protein flexibility that occurs during substrate binding.

**Figure 2:**
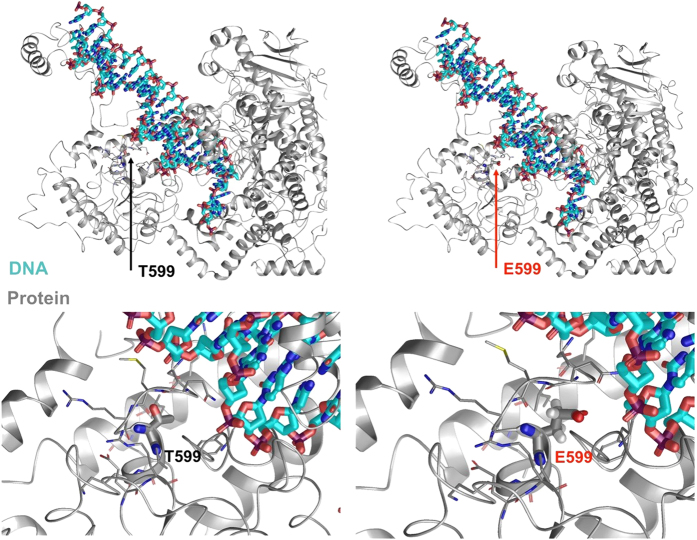
Schematic representation (three-dimensional molecular representation) of DNA polymerase gamma (POLG) side chain interactions within the local region and the T599E variant, as well as its effect on DNA enzymatic processes. DNA is shown with cyan carbons and POLG with gray carbons.

In addition, we modeled a different residue change at position 599, denoted p.T599E, to observe its effect and confirm that this conserved residue is important for wild-type POLG function. Molecular modeling of the p.T599E variant was performed, and Monte Carlo simulations suggested that the orientation of the T and E residues flip. The mutant may disrupt other POLG protein chains from binding and lead to changes in the helix–loop–helix conformation, where E599 is present in the middle of the loop (Figure 2). We found the energetic ΔΔ*G* shift to be −0.506754 kcal/mol Å^2^, which, from a modeling perspective, favors this variant over the wild type for the stabilization of local amino-acid residues.^13–15,17–20^

### Clinical description

A 42-year-old woman presented to our clinic with symptoms, including worsening gait instability, dysarthria and foot numbness of over a 1-year duration. She was first referred to our neurogenetics clinic after consulting several neurologists who were unable to determine an acquired cause for her symptomatology. Her neurologic examination revealed an ataxic dysarthria, restricted eye movements, inability to maintain fixation and head and palatal tremors. Her strength was normal, but she had reduced lower limb reflexes and distal multimodal sensory loss. Her gait was wide and unsteady, and she was unable to stand without support.

**Figure 3:**
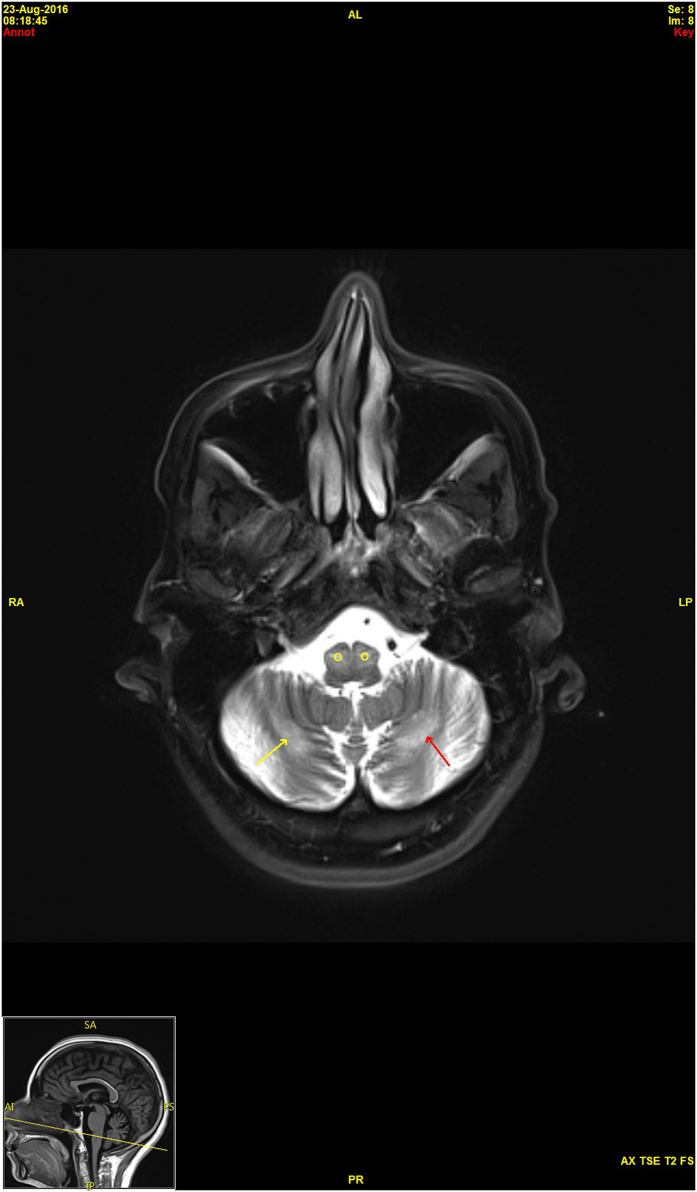
Axial T2 magnetic resonance imaging brain scan demonstrates bilateral, symmetrical hyperintense, hypertrophic olivary degeneration (O) and symmetrical bands of hyperintensity (arrows) in the cerebellar hemispheres.

Electromyography revealed reduced or absent sensory responses and normal motor responses. Her concentric needle examination was consistent with a sensory neuropathy or neuronopathy. A brain magnetic resonance imaging scan showed hypertrophic olivary degeneration and T2 hyperintense signal abnormalities in the cerebellar hemispheres, thalami and bilateral basal ganglia (Figure 3). Spinal cord imaging was unremarkable. Negative laboratory studies included a complete blood count, chemistry panel, vitamin B12, sedimentation rate, hemoglobin A1c, creatine kinase, thyroid profile, antinuclear antibody, extractable nuclear antigen evaluation, angiotensin-converting enzyme, serum protein electrophoresis, Lyme, serologies for HTLV I and II, and HIV, and *Tropheryma whipplei* PCR. A cerebrospinal fluid examination was normal, including cell count and protein and glucose levels, and the patient was negative for oligoclonal bands. Her electroencephalogram was normal. Following these tests, the patient was referred to neurogenetics where whole-exome sequencing and mtDNA analysis were ordered due to the suspected mitochondrial disease.

WES results indicated that the patient was homozygous for a variant of unknown significance, c.1795 A>C (p.T599P), in exon 10 of *POLG*. Because this substitution is non-conservative and alters the polarity of amino-acid residue 599, *in silico* prediction software showed that it likely damages the structure of the POLG protein. Using the PolyPhen-2 bioinformatics tools, the T599P variant was predicted to be “probably damaging”, with a score of 0.999. PolyPhen-2 predicts the effect of a single amino-acid substitution on the function of human proteins.^21^

**Figure 4:**
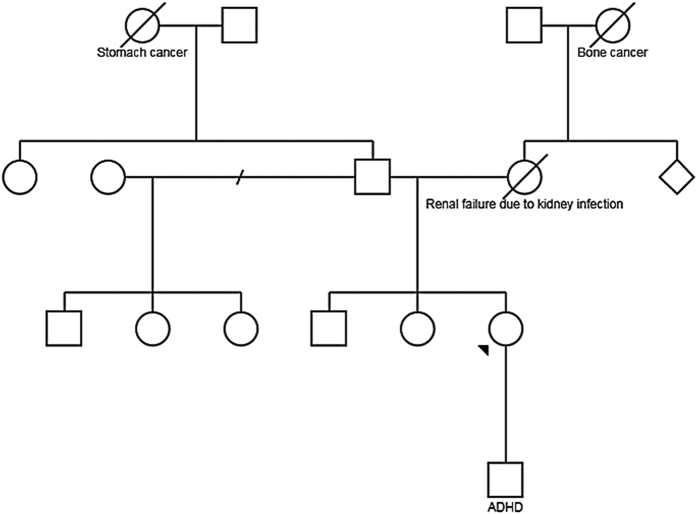
Family pedigree. The proband is indicated with an arrowhead.

In addition, no causative variants in disease genes associated with the patient’s phenotype were found, and mtDNA testing was normal. Negative *POLG* deletion and duplication tests, as well as an absence of the condition in other family members (Figure 4), indicated that the variant was novel and caused an autosomal recessive mitochondrial disease with the SANDO phenotype.

## Discussion

*POLG* pathogenic variants have heterogeneous phenotypes due to the vital importance of POLG, the only enzyme capable of replicating and repairing mitochondrial DNA in humans. If an individual is unable to produce wild-type POLG, respiratory chain complex I, III, IV and V dysfunction occurs, as a number of proteins in each complex is encoded by mtDNA. Mutant POLG often displays reduced mtDNA replication, leading to deletions or depletions of the mtDNA required to produce the complexes. Incomplete respiratory chain complexes inhibit proper ATP synthesis and cause a deficiency in energy metabolism.^4^ This, in turn, produces a variety of disease phenotypes, of which SANDO is only one example.^2^ SANDO remains a rare and challenging diagnosis, demonstrated in a small subset of 20+ patients since it was first described in 1997.^3^

Our particular patient has a novel pathogenic variant in *POLG*, which adds to the variant spectrum of this condition. This variant is not present in the Exome Aggregation Consortium database^22^ in either a heterozygous or homozygous state, demonstrating its uncommon nature in humans. Despite the uniqueness of this variant, it appears to result in a rather pedestrian SANDO symptomatology. The proband displays the classic symptoms of sensory ataxia neuropathy, dysarthria and ophthalmoplegia. The magnetic resonance imaging brain scan revealed signs of encephalopathy, a known symptom of the phenotype, but curiously, the proband did not present a history of seizures that often accompanies such degeneration. It is possible that the seizures will appear in time, considering the relatively short history of symptoms our proband has experienced. Aside from this small deviation, she appears to present the classic SANDO phenotype.^2^

Interestingly, p.T599P is the sole pathogenic variant found in our patient and lies in the linker domain of POLG. This domain has only been reported as causing SANDO without accompanying exonuclease or polymerase domain pathogenic variants in a few patients with the well-recognized p.A467T pathogenic variant.^23,24^ When one considers the high conservation rate of the linker domain and that its pathogenic variants cause phenotypes including the often fatal Alpers–Huttenlocher syndrome, the harmful nature of this new variant is not surprising.^25^ Overall, our investigation supports the concept that linker domain pathogenic variants are as capable of causing the usual symptoms of SANDO as the exonuclease or polymerase domain pathogenic variants.

Molecular modeling of the p.T599P variant was performed, and Monte Carlo simulations suggest that the introduction of proline induces a backbone dihedral adjustment of 135°. This adjustment significantly alters the orientation of the helix–loop–helix motif that is immediately adjacent to binding DNA (Figure 1). These conformational changes in the loop affect helix interactions with P599 and the adjacent DNA nucleotides that are effectively displaced. The energetics calculations show that the directly neighboring residues are further affected by the p.T599P variant compared with the wild type. The impact is twofold: there is an increased rigidity with the helix–loop–helix motif due to the double prolines at 599 and 600, and the side chains that have altered conformations (most importantly the Q595 and F766) are adjacent to DNA nucleotides under increased van der Waals forces, which may decrease nucleotide-binding efficiency.

Molecular modeling of the p.T599E variant suggests that the orientation of the T and E residues are flipped. These conformational changes in the loop affect helix interactions with E599 and the adjacent DNA nucleotides that have shifted. The energetics calculations show that the directly neighboring residues are further stabilized with the p.T599E variant compared to the wild type. This change may increase the rigidity between the helix–loop–helix motif, which increases the van der Waals forces on the adjacent DNA nucleotides, resulting in the slight nucleotide deviation observed. The extra rigidity in the local 6 Å region may alter the function of the flexible loop segment.

These molecular data demonstrate the importance of T599 in wild-type POLG function and support the observation that this residue is highly conserved across species.

There was a lengthy diagnostic odyssey involving multiple specialists at different institutes before a definite cause was determined for the patient’s condition. The hunt for an acquired cause of her symptoms resulted in multiple expensive and invasive tests (such as lumbar puncture), which may have been avoided with early genetic testing. This case highlights that whole-exome sequencing is a valuable tool by which new pathogenic variants can be identified, as this form of testing revealed the patient’s condition. We demonstrate that if a novel variant of unknown significance is identified, we can perform neoteric functional protein modeling to evaluate the effect on protein dynamics and subsequently use this information to aid in a clinical diagnosis. In addition, we demonstrate the importance of considering SANDO when patients present the triad symptoms of sensory neuropathy, dysarthria and ophthalmoparesis. In similar cases, whole-exome sequencing could be performed first to truncate costs and time between presentation and diagnosis when this difficult constellation of symptoms is recognized.

In summary, we demonstrate a novel protein modeling method for variant interpretation of a *POLG* variant of unknown significance that can be expanded and used for other genetic conditions and protein variants. In addition, this process can be used to determine why particular protein residues are conserved across species.

### Availability of data and materials

Data sets and materials are detailed in the manuscript.

## Publisher's Note

Springer Nature remains neutral with regard to jurisdictional claims in published maps and institutional affiliations.
